# Mitochondrial genome editing: Get aCcess to modeling broad disease mutations with engineered base editors

**DOI:** 10.1016/j.omtn.2023.04.014

**Published:** 2023-05-05

**Authors:** Chunlong Xu, Pengyu Huang

**Affiliations:** 1 Lingang Laboratory, Shanghai 200031, China; 2 Shanghai Research Center for Brain Science and Brain-Inspired Intelligence, Shanghai 200031, China; 3 Institute of Biomedical Engineering, Chinese Academy of Medical Sciences and Peking Union Medical College, Tianjin 201210, China; 4 Tianjin Institutes of Health Science, Tianjin 301600, China

As the major energy suppliers in our cells, mitochondria play important roles in both physiological and pathological processes. Unlike other cellular organelles, mitochondria have their own circular genome, comprising several genes indispensable for normal mitochondrial functions. Genetic mutations in mitochondrial DNA (mtDNA) can cause various fatal diseases and disorders in a maternally inherited manner. However, it has been difficult to generate animal models for complex mitochondrial diseases due to the lack of gene editing tools for precise modification of mtDNA.^1^ Several years ago, David Liu and colleagues developed a CRISPR-free base editor, DdCBE, that enabled the first introduction of C-to-T base mutations in mtDNA.^2^ However, this canonical DdCBE could not edit mtDNA sequences without a 5′-tC-3′ motif,^3^ accounting for less than 30% of C:G>T:A mutations identified from patients with mitochondrial diseases. Nevertheless, more than 70% of C:G>T:A mutations carry 5′-aC-3′, 5′-cC-3′, or 5′-gC-3′ motifs. In this issue of *Molecular Therapy – Nucleic Acids*, Qi et al.^4^ engineered canonical DdCBE to edit mtDNA within a broad sequence context. The authors achieved successful modification of several 5′-aC-3′ sequences and generated mitochondrial gene-edited rats with human-like symptoms.

By splitting deaminase at two different positions (G1333 and G1397 a.a.) of DddA proteins, Qi et al. engineered different pairs of TALE-DddA fusion proteins and tested them with 11 mtDNA loci corresponding to human pathogenic mutations carrying 5′-aC-3′, 5′-cC-3′, or 5′-gC-3′ motifs in rat glioma cells. They showed the L1333C + R1333N combination exhibited robust base editing activity for 5′-aC-3′ sequences but only marginal activity for 5′-cC-3′ sequences. Unfortunately, none of the split TALE-DddA combinations induced appreciable C-to-T editing for 5′-gC-3′ sequences.

All selected mtDNA loci tested in this study are pathogenic and conserved between rat and human mitochondrial genomes. Therefore, the authors injected DdCBE pairs for editing 5′-aC-3′ into rat zygotes. This generated F0 animals carrying different loads of mtDNA mutations. Rats carrying mtDNA mutations for G007A and G11714/5A developed dilated cardiomyopathy (DCM) and hypertrophic cardiomyopathy (HCM), respectively. The authors also found that mutant female rats transmitted the mutations to over 80% of their offspring. For the G007A mutation, obvious DCM manifested in F1 pups as well, which recapitulates the phenotype in human patients. Intriguingly, G11714/5A-driven HCM has not been documented in human cases, suggesting that patients carrying homologous mutations might also be susceptible to HCM. Thus, these animal models may contribute to our understanding of human mitochondrial diseases and even to developing treatment for disease intervention.

The pathogenic mtDNA mutations introduced in rat by Qi et al. arose from genes encoding different tRNAs in mitochondria. These tRNA mutations reduce global protein translation in mitochondria, dramatically impairing normal mitochondrial morphology and functions. Since muscle and heart are high-energy-demanding tissues, mitochondrial dysfunction caused by tRNA mutations leads to muscle weakness and cardiomyopathy in rat. Interestingly, the authors found a significant increase in mtDNA transcription and copy number in mutant tRNA-bearing rat tissues, which might represent a general feedback mechanism underlying mitochondrial diseases.

The present study demonstrated the excellent performance of DdCBE engineering for complex mtDNA editing for modeling mitochondrial disease. To further increase editable sequences for DdCBE, two other groups have recently developed novel DdCBE tools via protein evolution^3^ and DddA ortholog screening^5^ for C-to-T editing on cytosine within the 5′-aC-3′, 5′-cC-3′, and 5′-gC-3′ motifs. Moreover, Cho et al.^6^ achieved mitochondrial A-to-G editing, further diversifying the mtDNA editing toolkit.

Altogether, these findings may facilitate mitochondrial disease modeling and treatment using DdCBEs. Nevertheless, substantial off-target effects independent of Transcription activator-like effector (TALE) binding specificity were previously reported for DdCBE,^7^^,^^8^ and this issue was not resolved in Qi and colleagues’ study. These potential off-target effects warrant stringent evaluation of the TALE-independent off-target profile in the future when mitochondrial disease treatment ushers in 5′-aC-3′ editing via DdCBE. At present, an engineered DdCBE^8^^,^^9^ with efficient editing activity for broad sequence specificity and minimal off-target effects remains an unmet need.
